# Measuring Adipocytokines in Young Patients With Type 2 Diabetes Presenting to a Tertiary Care Centre in Upper Assam, India

**DOI:** 10.7759/cureus.98779

**Published:** 2025-12-09

**Authors:** Anupam Dutta, Meenakshi Saikia, Pranjal K Dutta, Sanjeeb Kakati, Angshuman Boruah, Neelakshi Bhattacharyya, Rahul Neog, Reema Nath

**Affiliations:** 1 Department of General Medicine, Assam Medical College and Hospital, Dibrugarh, IND; 2 Department of Biochemistry, Assam Medical College and Hospital, Dibrugarh, IND; 3 Department of Internal Medicine, Assam Medical College and Hospital, Dibrugarh, IND; 4 Multidisciplinary Research Unit, Assam Medical College and Hospital, Dibrugarh, IND; 5 Department of Microbiology, Assam Medical College and Hospital, Dibrugarh, IND

**Keywords:** adipocytokines, adiponectin, c-reactive protein, il-6, inflammation, lean diabetes, leptin, northeast india, phenoeindy-2

## Abstract

Background

Type 2 diabetes mellitus (T2DM) in lean and young adults is emerging as a distinct phenotype in South Asia, often dissociated from obesity-driven insulin resistance. This study evaluated adipocytokines (adiponectin and leptin) and inflammatory markers (C-reactive protein (CRP) and interleukin-6 (IL-6)) in young T2DM and non-diabetic individuals across body mass index (BMI) categories to elucidate underlying metabolic and inflammatory profiles.

Methods

A hospital-based case-control analysis was conducted among 176 individuals (age and sex matched, 83 diabetics, 93 non-diabetics) aged 18-45 years (105 females, 71 males), drawn from the PHENOEINDY-2 cohort at Assam Medical College and Hospital, Dibrugarh. Participants were stratified into four BMI groups: underweight (<18.5 kg/m²), normal (18.5-24.9 kg/m²), overweight (25.0-29.9 kg/m²), and obese (>30 kg/m²). Serum adiponectin, leptin, IL-6, and high-sensitivity CRP (hs-CRP) were measured using enzyme-linked immunosorbent assay (ELISA)-based assays. Statistical comparisons employed t-tests, ANOVA, and regression modeling, adjusting for age and socioeconomic factors.

Results

Among diabetics, mean adiponectin was highest in the underweight group (3.44 ± 2.56 µg/mL) and declined with increasing BMI, showing a significant negative correlation with BMI (r = -0.65, P = 0.079). Leptin demonstrated a strong positive correlation with BMI (r = 0.77, P = 0.024), increasing progressively from underweight (0.54 ± 0.66 ng/mL) to obese participants (0.80 ± 0.96 ng/mL). CRP levels were markedly elevated in underweight diabetics (8.15 ± 11.9 mg/L) versus non-diabetics (2.19 ± 1.75 mg/L, P = 0.075), suggesting low-grade inflammation even in non-obese individuals. A positive association between CRP and BMI (r = 0.67, P = 0.012) was observed overall. IL-6 levels peaked among normal-weight (102.36 ± 179.5 pg/mL) and overweight diabetics (118.72 ± 163.38 pg/mL), without significant correlation with BMI (r = -0.12, P = 0.777), indicating adiposity-independent inflammation. Logistic regression incorporating all biomarkers yielded a modest predictive accuracy of 33.3%, with CRP emerging as the most influential predictor of diabetes risk, followed by IL-6. Scatter plots confirmed negative trends for adiponectin and positive associations for leptin and CRP with BMI, while IL-6 remained BMI-independent.

Interpretation

Lean diabetics exhibited distinct biochemical features - higher adiponectin but elevated CRP and IL-6 - supporting inflammation and β-cell dysfunction as key drivers over insulin resistance. In contrast, obese diabetics demonstrated low adiponectin and high leptin, reflecting adiposity-linked metabolic stress. The elevated inflammatory markers among lean diabetics highlight non-adipose origins of inflammation, possibly from hepatic or immune pathways.

Conclusions

This study underscores heterogeneity in T2DM pathophysiology across BMI categories in Northeast India. Inflammation, rather than adiposity, may underlie dysglycemia among lean individuals, while adipokine imbalance predominates in obesity-associated diabetes. Integrating adiponectin, CRP, and IL-6 into risk assessment could refine screening for atypical or lean T2DM phenotypes. Future longitudinal and genetic studies are warranted to dissect the contribution of developmental and ethnic factors unique to this population.

## Introduction

Type 2 diabetes mellitus (T2DM) is a multifactorial disease influenced by complex interactions between genetic, environmental, and metabolic factors. Globally, the burden of T2DM continues to rise, disproportionately affecting low- and middle-income countries, including India [1]. Of particular concern is the increasing incidence of T2DM in young adults and in individuals with a body mass index (BMI) within the normal or underweight range. This phenomenon, often termed “lean diabetes,” challenges the classical paradigm that links T2DM predominantly to obesity and insulin resistance [2].

In the Indian subcontinent, and more specifically in the ethnically diverse region of Northeast India, young-onset and lean T2DM are increasingly reported. The underlying mechanisms remain poorly understood but are believed to extend beyond traditional adiposity-driven insulin resistance [3]. Emerging evidence suggests that chronic low-grade inflammation and dysregulation of adipose-derived cytokines, or adipokines, may play critical roles in the pathogenesis of T2DM even in non-obese individuals [4].

Adiponectin and leptin are two major adipokines with contrasting metabolic roles. Adiponectin possesses anti-inflammatory, insulin-sensitizing, and anti-atherogenic properties. Low adiponectin levels have been associated with increased risk of T2DM and cardiovascular disease, especially in populations with lower BMI [5]. Leptin, on the other hand, regulates satiety and energy expenditure but can promote insulin resistance in the setting of hyperleptinemia and leptin resistance, particularly in obesity [6]. However, its role in lean diabetes remains ambiguous.

Inflammatory cytokines such as interleukin-6 (IL-6) and C-reactive protein (CRP) have been consistently implicated in the early pathophysiological changes of T2DM. Elevated IL-6 levels can directly impair pancreatic β-cell function and increase hepatic glucose production, while high CRP levels indicate systemic inflammation, which contributes to insulin resistance [7]. Notably, these markers may be elevated even in the absence of obesity, suggesting that inflammation could serve as an independent risk factor in lean diabetes [8].

The pathophysiology of lean T2DM in South Asians may be further complicated by developmental, genetic, and socioeconomic influences. Poor fetal and early childhood nutrition - prevalent in many parts of rural India - may lead to a “thin-fat” phenotype characterized by visceral adiposity and metabolic dysfunction despite a normal BMI [9]. Studies also suggest that epigenetic modifications and intergenerational undernutrition may predispose individuals to β-cell dysfunction and inflammatory metabolic programming [10].

Despite these insights, there is a paucity of data on inflammatory and adipokine profiles among lean T2DM individuals in Northeast India. Most available studies focus on obese or overweight diabetics, leaving a significant knowledge gap regarding non-obese diabetics, especially among ethnically diverse and nutritionally vulnerable populations. Understanding the interplay between adiponectin, leptin, CRP, and IL-6 in this unique demographic may yield important insights into the pathogenesis of lean diabetes.

This study, therefore, aims to evaluate the levels of adiponectin, leptin, CRP, and IL-6 in diabetic and non-diabetic individuals across BMI categories in a subset of participants from the PHENOEINDY-2 cohort in Upper Assam [11]. By analyzing these biomarkers, we seek to identify distinct inflammatory and metabolic patterns associated with lean T2DM, and to explore the utility of these biomarkers in refining risk assessment and potential therapeutic targeting for this under-recognized subtype of diabetes.

## Materials and methods

This hospital-based, case-control study was conducted at the Diabetes Research Unit of Assam Medical College and Hospital (AMCH), Dibrugarh, Assam, India. The study population comprised a subset of participants from the PHENOEINDY-2 cohort, a previously conducted research initiative focused on young-onset type 2 diabetes mellitus (T2DM) in Northeast India. A total of 176 individuals were enrolled in the present study, including 83 diabetic (International Diabetes Federation criteria) and 93 non-diabetic participants (healthy acquaintances like friends and family of diabetic patients, from the same socioeconomic and ethnic background). These individuals were stratified into four body mass index (BMI) categories based on World Health Organization (WHO) criteria: underweight (BMI <18.5 kg/m²), normal weight (BMI 18.5-24.9 kg/m²), overweight (BMI 25.0-29.9 kg/m²), and obese (BMI >30 kg/m²).

Participants were selected based on specific inclusion and exclusion criteria. The inclusion criteria comprised: (i) diagnosed cases of T2DM in accordance with WHO/International Diabetes Federation (IDF) diagnostic criteria, defined as fasting plasma glucose levels greater than 126 mg/dL, postprandial plasma glucose exceeding 200 mg/dL, or glycated hemoglobin (HbA1c) levels above 6.5%; (ii) age between 18 and 45 years at the time of diagnosis; and (iii) willingness to provide informed written consent. Individuals were excluded from the study if they had a diagnosis of Type 1 diabetes (using C-peptide), Maturity Onset Diabetes of the Young (MODY), or secondary diabetes using proper history, clinical examination, and relevant laboratory tests. Additional exclusion criteria included a history of acute infections, chronic inflammatory diseases, malignancies, alcohol use, smoking, or recent use of corticosteroids.

Each participant underwent a comprehensive clinical and anthropometric assessment. Measurements included height, weight, BMI, waist-hip ratio (WHR), and mid-arm circumference. Blood pressure was measured in a seated position following a 10-minute rest period using a calibrated sphygmomanometer.

For biochemical analyses, fasting venous blood samples were collected from all participants after an overnight fast of 8-12 hours. In the non-diabetic control group, an oral glucose tolerance test (OGTT) was performed, and only individuals with normal glucose tolerance were included in the study. Lipid biomarker assays were conducted as follows: adiponectin and leptin (samples collected and stored in multiple aliquots in a −80 ℃ refrigeratorin our bio-bank) levels were estimated using enzyme-linked immunosorbent assay (ELISA) kits sourced from Diagnostic Biochem Canada (London, Ontario, Canada). C-reactive protein (CRP) levels were measured using a high-sensitivity CRP (hs-CRP) assay, while interleukin-6 (IL-6) concentrations were quantified using commercial ELISA kits from R&D Systems (Minneapolis, USA).

Data were statistically analyzed using Stata software version 15.1 (StataCorp, College Station, USA). Continuous variables were expressed as mean ± standard deviation (SD) or as median with interquartile range (IQR), depending on the data distribution. Categorical variables were presented as percentages. For comparisons between diabetic and non-diabetic groups, independent t-tests or Mann-Whitney U tests were used for continuous variables, while Chi-square tests were applied to assess differences in categorical data. Analysis of variance (ANOVA) was employed for comparing multiple groups across BMI categories, with adjustments made for age and socioeconomic factors (ANCOVA). Additionally, logistic regression models were constructed to evaluate the predictive value of adiponectin, leptin, CRP, and IL-6 for diabetes risk, with results reported as odds ratios (ORs) along with 95% confidence intervals (CIs). A P-value of less than 0.05 was considered statistically significant.

This study received approval from the Institutional Ethics Committee of Assam Medical College (approval no. 2022/AMC/EC/1932 dated 26th August 2022). Written informed consent was obtained from all participants prior to inclusion. Confidentiality of participant data was strictly maintained, and all procedures were conducted in accordance with the ethical principles outlined in the Declaration of Helsinki (2013 revision).

## Results

The study analyzed 176 individuals, comprising 83 diabetics and 93 non-diabetics, stratified across four BMI categories: underweight (<18.5 kg/m², n=37), normal (18.5-24.9 kg/m², n=50), overweight (25.0-29.9 kg/m², n=49), and obese (>30 kg/m², n=40). Diabetic participants were consistently older than their non-diabetic counterparts across all BMI strata. A rural predominance was noted among underweight and normal-weight individuals, while overweight and obese categories showed an urban skew in both diabetic and non-diabetic groups (Table 1). 

**Table 1:** Summary of biomarker levels (median with 25th to 75th centile) in diabetics and non-diabetics across BMI catagories Values are expressed as median with 25th and 75th centiles. P-values were calculated using independent t-tests. None of the comparisons reached statistical significance (P < 0.05), but trends were observed. CRP: C-reactive protein

Biomarker	BMI Category	Diabetics (n=83) Median (25th - 75th centile)	Non-diabetes (n=93) Median (25th - 75th centile)	P value
Adiponectin (µg/mL)	Underweight	3.07 (1.21-6.13)	1.13 (0.67-1.71)	0.181
	Normal	1.03 (0.62-2.33)	1.06 (0.68-1.79)	0.422
	Overweight	0.56 (0.44-0.74)	1.19 (0.51-1.62)	>0.05
	Obese	0.91 (0.44-1.24)	0.92 (0.33-1.58)	>0.05
Leptin (ng/mL)	Underweight	0.46 (0.37-0.59)	0.51 (0.41-0.57)	0.756
	Normal	0.44 (0.34-0.81)	0.56 (0.43-1.01)	0.818
	Overweight	0.59 (0.49-1.64)	0.78 (0.38-1.81)	>0.05
	Obese	0.50 (0.36-1.01)	0.95 (0.30-2.28)	0.132
CRP (mg/L)	Underweight	2.81 (2.27-10.7)	1.79 (1.34-2.33)	0.075
	Normal	5.60 (2.04-11.52)	3.09 (1.78-5.96)	0.113
	Overweight	4.8 (2.50-11.94)	4.76 (3.75-9.25)	0.634
	Obese	6.61 (5.08-22.2)	13.80 (4.90-19.85)	0.634
IL-6 (pg/mL)	Underweight	16.8 (5.01-28.15)	24.10 (11.30-61.0)	0.624
	Normal	31.5 (23.8-58.5)	25.5 (11.4-45.6)	0.102
	Overweight	47.5 (24.2-113.0)	24.6 (21.0-41.7)	0.182
	Obese	20.25 (5.65-56.9)	32.6 (15.8-49.0)	0.340

Adiponectin levels

Adiponectin concentrations were highest in underweight diabetics (median 3.07 µg/mL) compared to underweight non-diabetics (median 1.13 µg/mL), although this difference was not statistically significant (P = 0.181). A consistent trend of declining adiponectin levels with increasing BMI was observed across both diabetic and non-diabetic groups, with the lowest adiponectin levels for diabetics recorded in the overweight group and in non-diabetics in the normal group. Regression analysis demonstrated a moderately strong negative correlation between BMI and adiponectin levels (r = -0.65, P = 0.079), indicating that adiponectin diminishes with increasing adiposity regardless of diabetic status (Table 1).

Leptin levels

Leptin levels showed a predictable positive correlation with BMI (r = 0.77, P = 0.024), affirming its role as a surrogate marker of adiposity. The highest leptin concentrations were observed in the obese group, amongst non-diabetics, but in diabetics, leptin was the lowest amongst the obese subgroup. However, no statistically significant differences in leptin levels were found between diabetics and non-diabetics across any BMI category. In the obese subgroup, median leptin was higher in non-diabetics (0.95 ng/mL) than diabetics (0.50 ng/mL), but the difference was not significant (P = 0.132).

C-reactive protein (CRP) levels

C-reactive protein, a marker of systemic inflammation, displayed elevation in underweight, normal weight, and overweight diabetics compared to their non-diabetic counterparts; however, in obese patients, it was the non-diabetics who showed higher results. Across other BMI categories, CRP levels were elevated in both diabetic and non-diabetic individuals, but the differences did not reach statistical significance. A strong positive correlation was found between CRP and BMI (r = 0.67, P = 0.012), supporting its association with obesity-related inflammation. However, the elevated CRP levels in underweight diabetics suggest that inflammation may also play a role in non-obese forms of diabetes.

Interleukin-6 (IL-6) levels

**Figure 1:**
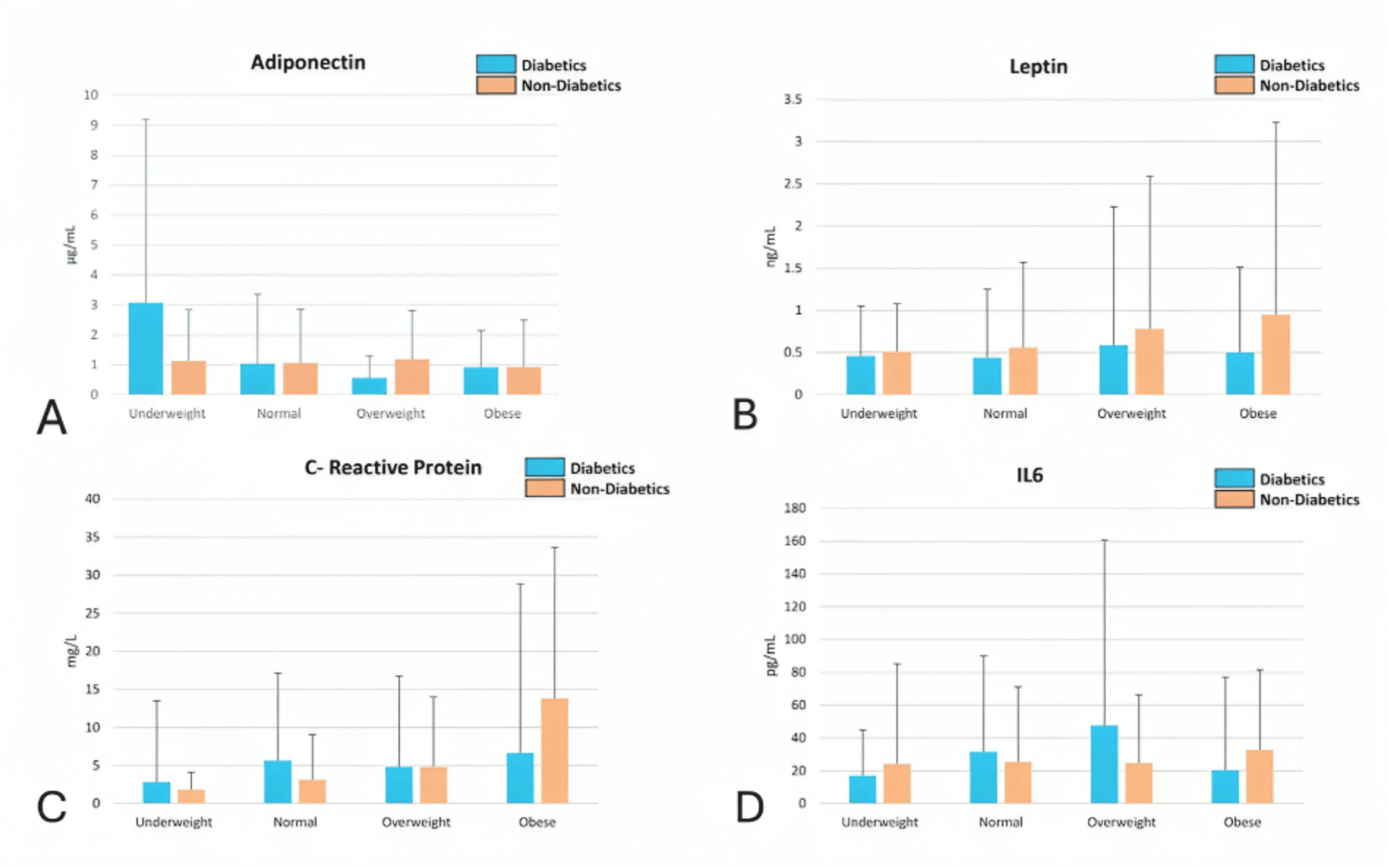
Graphical representation of adiponectin, leptin, CRP, and IL-6 levels across different BMI categories for diabetic and non-diabetic individuals Comparison of adipokines and inflammatory markers across BMI categories in diabetic and non-diabetic groups. (A) Adiponectin: Levels are higher among underweight diabetics compared to non-diabetics, with a declining trend as BMI increases in both groups. (B) Leptin: Leptin concentrations rise progressively from underweight to obese categories, more prominently among non-diabetics. (C) C-Reactive Protein (CRP): CRP shows higher values in diabetics across all BMI groups except in the obese, where it is higher in non-diabetics, indicating enhanced systemic inflammation. (D) Interleukin-6 (IL-6): IL-6 levels are elevated in both groups, with diabetics showing a relatively higher mean concentration, especially in the overweight category.

Interleukin-6 levels were highest in normal-weight (median 31.5 pg/mL) and overweight diabetics (mean 47.5 pg/mL). Interestingly, IL-6 was not significantly elevated in obese diabetics and did not correlate meaningfully with BMI (r = -0.12, P = 0.777), suggesting that IL-6-associated inflammation may be independent of adiposity in this population. In underweight individuals, non-diabetics exhibited higher IL-6 levels (median 24.1 pg/mL) than diabetics (median 16.8 pg/mL), possibly reflecting environmental stress or malnutrition-related inflammation (Figure 1).

Predictive modeling and regression analysis

**Figure 2:**
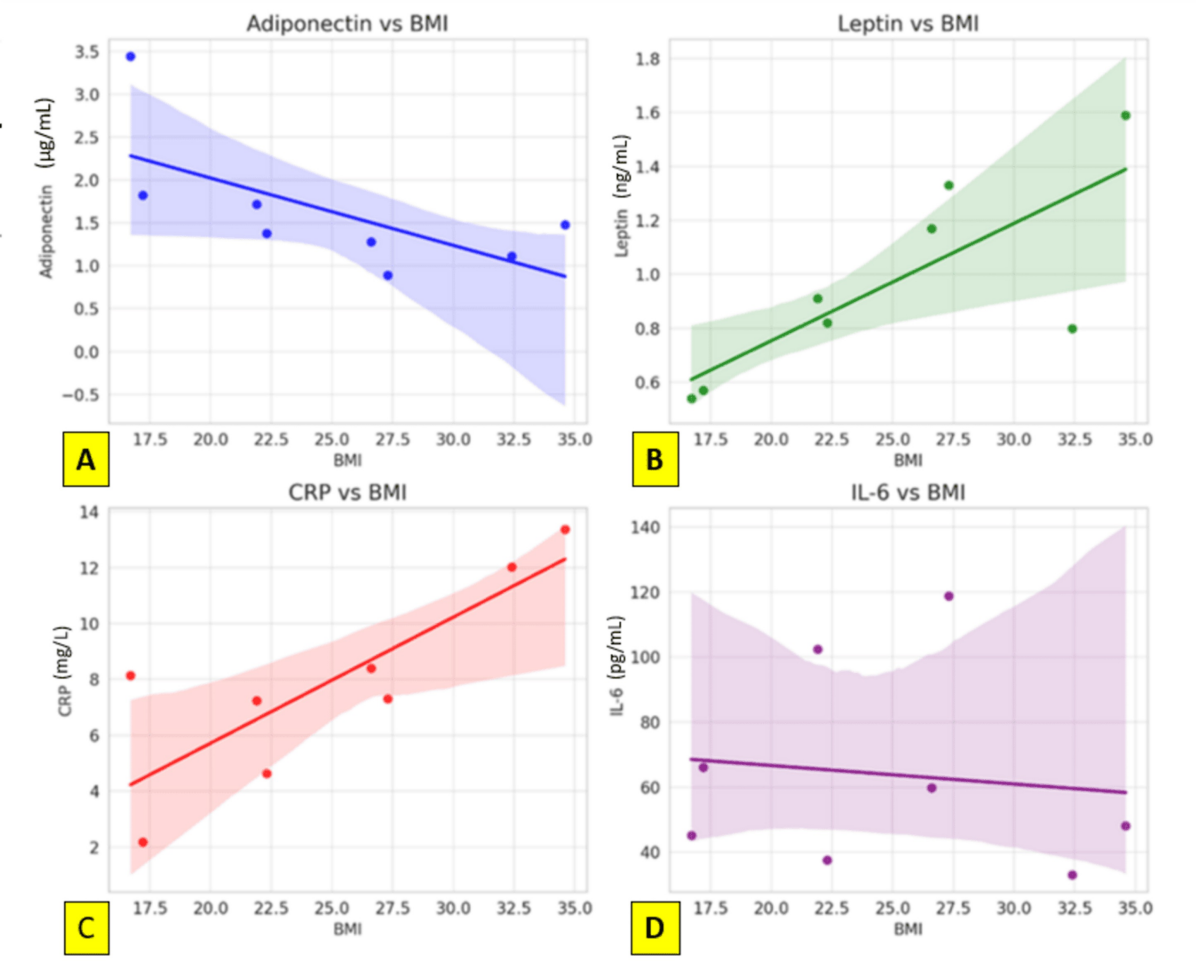
Scatter plots with regression lines for adiponectin, leptin, CRP, and IL-6 vs. BMI to visualize trends, along with the results of statistical modeling Relationship of adipokines and inflammatory markers with BMI. (A) Adiponectin vs BMI: A negative correlation is observed, indicating lower adiponectin levels with increasing BMI. (B) Leptin vs BMI: Leptin levels show a positive association with BMI, increasing with higher adiposity. (C) CRP vs BMI: C-reactive protein demonstrates a positive trend, suggesting higher systemic inflammation with increasing BMI. (D) IL-6 vs BMI: IL-6 levels show a mild, non-significant inverse relationship with BMI, indicating variable inflammatory responses across BMI categories.

A logistic regression model including adiponectin, leptin, CRP, and IL-6 as independent predictors showed limited discriminatory power, with an overall predictive accuracy of only 33.3%. Among the markers analyzed, CRP demonstrated the strongest predictive value for diabetes, followed by IL-6. Adiponectin and leptin were not significant independent predictors in this model (Figure 2).

Correlation summary and visual trends

Scatter plots confirmed the observed trends. Adiponectin showed a negative correlation with BMI (R² = 0.43), leptin showed a strong positive correlation (R² = 0.60), and CRP also exhibited a significant positive relationship with BMI (R² = 0.67). Conversely, IL-6 did not display a significant trend with BMI (R² = 0.014), reinforcing its role as an obesity-independent inflammatory marker.

## Discussion

This study provides valuable insights into the metabolic and inflammatory differences between lean and obese individuals with type 2 diabetes mellitus (T2DM) in Northeast India. The findings emphasize that lean diabetics demonstrate a distinct biochemical and pathophysiological profile that may not conform to the classical insulin resistance-driven model seen in obesity-related diabetes. The altered levels of adipokines such as adiponectin and leptin, along with pro-inflammatory markers like CRP and IL-6, underscore the complexity of diabetes etiology in this population.

CRP and IL-6: evidence of inflammation in lean diabetes

C-reactive protein (CRP), a widely recognized marker of systemic inflammation, was found to be significantly elevated in underweight diabetics compared to their non-diabetic counterparts. This finding is consistent with studies indicating that inflammation plays a critical role in the pathogenesis of diabetes, even in individuals with low BMI. For instance, Pradhan et al. demonstrated that elevated baseline levels of CRP and IL-6 were predictive of future diabetes in apparently healthy individuals, independent of BMI or obesity status [12]. Similarly, the European Prospective Investigation into Cancer and Nutrition (EPIC)-Potsdam study reported that higher levels of IL-6 and CRP were associated with an increased risk of T2DM in a European cohort, reinforcing the inflammatory hypothesis of diabetes [8].

In our study, IL-6 levels were notably higher in normal-weight and overweight diabetics compared to their non-diabetic counterparts. This supports the notion that IL-6 may impair β-cell function and insulin signaling, contributing to hyperglycemia in the absence of significant obesity. IL-6 has also been shown to induce hepatic gluconeogenesis and reduce insulin receptor signaling in peripheral tissues [13]. Importantly, the weak correlation of IL-6 with BMI in this study suggests that the source of inflammation in lean diabetics may not be adipose tissue alone but could involve non-adipose sites such as the liver, gut, or immune system.

Adiponectin: a marker of insulin sensitivity and β-cell function

Adiponectin, an adipokine with potent insulin-sensitizing and anti-inflammatory properties, showed an inverse relationship with BMI and was significantly higher in underweight diabetics compared to obese diabetics. This aligns with previous findings that lean diabetics often have preserved or even elevated adiponectin levels, suggesting that their hyperglycemia may result more from β-cell dysfunction than from insulin resistance [14]. In contrast, obese diabetics, who typically have low adiponectin levels, are more likely to exhibit insulin resistance as the primary pathogenic mechanism [15].

These observations support the idea that lean diabetes may represent a distinct phenotype, particularly in South Asian populations where early-life undernutrition and epigenetic programming may impair pancreatic β-cell mass or function [3]. Moreover, the relatively high adiponectin levels in lean diabetics may partially protect against atherogenesis and metabolic syndrome, despite poor glycemic control.

Leptin: reflections of adipose mass rather than disease state

Leptin levels in this study were positively correlated with BMI but did not differ significantly between diabetic and non-diabetic individuals within the same BMI categories. This is in line with studies that view leptin primarily as a surrogate of fat mass rather than a direct mediator of insulin resistance or diabetes [16]. While extreme leptin deficiency, as seen in congenital lipodystrophy, is associated with severe insulin resistance and diabetes [17], our results suggest that leptin plays a minimal role in the pathogenesis of T2DM among the general population in this region.

Thus, while leptin may inform us about adiposity, it is unlikely to serve as a useful biomarker for distinguishing diabetics from non-diabetics, especially in lean individuals.

Ethnic and regional differences in diabetes phenotypes

South Asians, including those from Northeast India, develop T2DM at lower BMI levels and with less overt adiposity than their Western counterparts. Several studies have attributed this to increased visceral fat, reduced skeletal muscle mass, and genetic or epigenetic factors influencing glucose metabolism [18]. A seminal study by Chandalia et al. highlighted that Asian Indians had significantly higher insulin resistance than Caucasians at comparable BMI and body fat levels [19]. This is corroborated by Yajnik’s work on the “thin-fat” Indian phenotype, characterized by low BMI but high fat mass and increased metabolic risk [20].

These regional and ethnic differences necessitate a reevaluation of traditional diabetes screening criteria. Sole reliance on BMI may fail to identify at-risk lean individuals, especially in low-resource settings where advanced diagnostic tools are scarce.

Clinical and public health implications

The findings from our study have both diagnostic and therapeutic implications. First, biomarkers such as adiponectin, CRP, and IL-6 could be integrated into screening protocols to better identify individuals at risk of lean diabetes. Second, treatment strategies for lean diabetics may need to prioritize β-cell preservation and inflammation reduction, rather than focusing solely on improving insulin sensitivity. Anti-inflammatory agents targeting IL-6 or CRP pathways, currently being evaluated in other contexts, might hold promise for specific diabetic phenotypes [21,22,23].

Furthermore, public health interventions aimed at improving maternal and early-life nutrition could reduce the long-term risk of lean diabetes, particularly in under-resourced and rural communities.

Limitations and future directions

This study is limited by its hospital-based design, which may not fully capture the diversity of the general population. Moreover, while adipokines and inflammatory markers were assessed, genetic factors, lifestyle behaviors, and longitudinal outcomes were not evaluated. Future research should focus on population-based cohorts and incorporate genomic, epigenomic, and microbiome data to elucidate the multifactorial origins of lean diabetes.

## Conclusions

This study delineates a distinct biochemical and inflammatory profile among young type 2 diabetes mellitus (T2DM) patients in Upper Assam, underscoring the heterogeneity of diabetes across body mass index (BMI) categories. Lean diabetics demonstrated relatively higher adiponectin but elevated inflammatory markers, particularly CRP and IL-6, suggesting that chronic low-grade inflammation and β-cell dysfunction, rather than obesity-related insulin resistance, are central to disease pathogenesis in this subgroup. Conversely, obese diabetics exhibited low adiponectin and high leptin, consistent with adiposity-driven metabolic stress.

These findings reinforce that inflammation in lean diabetes is not strictly adipose-dependent and may arise from hepatic, immune, or environmental origins. The weak predictive power of individual biomarkers also highlights the multifactorial nature of diabetes in this ethnically diverse region.

Future research should pursue longitudinal cohort studies incorporating genomic, epigenetic, and proteomic analyses to clarify the mechanistic links between inflammation, β-cell dysfunction, and body composition. Evaluating early-life nutritional and developmental factors, coupled with regional genetic diversity, could further illuminate the origins of lean diabetes. Expanding biomarker panels to include novel inflammatory and endothelial mediators may improve disease prediction and inform precision-targeted interventions.

Ultimately, understanding these unique metabolic signatures can refine screening strategies, guide personalized therapy, and address the rising burden of non-obese diabetes in Northeast India.
